# Borneol Depresses P-Glycoprotein Function by a NF-κB Signaling Mediated Mechanism in a Blood Brain Barrier *in Vitro* Model

**DOI:** 10.3390/ijms161126051

**Published:** 2015-11-18

**Authors:** Xiang Fan, Lijuan Chai, Han Zhang, Yuefei Wang, Boli Zhang, Xiumei Gao

**Affiliations:** 1 Institute of Traditional Chinese Medicine, Tianjin University of Traditional Chinese Medicine, Tianjin 300193, China; cljuan1258@163.com (L.C.); zhanghan0023@126.com (H.Z.); wangyuefei_2006@hotmail.com (Y.W.); zhangbolipr@163.com (B.Z.); gaoxiumei@tjutcm.edu.cn (X.G.); 2 Tianjin State Key Laboratory of Modern Chinese Medicine, Tianjin University of Traditional Chinese Medicine, Tianjin 300193, China

**Keywords:** borneol, P-glycoprotein, brain microvascular endothelial cells, NF-κB, blood brain barrier

## Abstract

P-glycoprotein (P-gp) on brain microvascular endothelial cells (BMECs) that form the blood brain barrier (BBB), influences transportation of substances between blood and brain. The objective of this study was to characterize the effects of borneol on P-gp efflux function on BBB and explore the potential mechanisms. We established an *in vitro* BBB model comprised of rat BMECs and astrocytes to measure the effects of borneol on the known P-gp substrates transport across BBB, and examined the function and expression of P-gp in BMECs and the signaling pathways regulating P-gp expression. Borneol increased intracellular accumulation of Rhodamine 123, enhanced verapamil and digoxin across the BBB *in vitro* model, and depressed mdr1a mRNA and P-gp expression. Borneol could activate nuclear factor-κB (NF-κB) and inhibition of NF-κB with MG132 (carbobenzoxy-Leu-Leu-leucinal) and SN50 (an inhibitory peptide) obscuring the P-gp decreases induced by borneol. These data suggested that borneol depresses P-gp function in BMECs by a NF-κB signaling medicated mechanism in a BBB *in vitro* model.

## 1. Introduction

The blood brain barrier (BBB) consisting of brain microvascular endothelial cells (BMECs) sealed together by continuous tight junctions plays a pivotal role to control the transportation of substances from blood to brain parenchyma and maintain brain microenvironment homeostasis [1]. P-glycoprotein (P-gp) and multidrug resistance-associated proteins have been shown to be expressed in BMECs, which can transport many physiological and pharmacological substances from the brain to the blood [2,3,4]. Transport medicated by these efflux pumps is important with respect to central nervous system drug clearance and limits drug delivery into the brain.

P-gp, a 170 kDa glycoprotein, is one of the ATP-binding cassette (ABC) superfamily of membrane transporters and is encoded by multidrug resistance (MDR) genes [2,5]. Human MDR1 together with rodent mdr1a and mdr1b selectively are identified with multidrug resistance. P-gp is an energy-dependent efflux pump and can transport a wide range of substances including morphine, phenytoin, flesinoxan, anti-cancer drugs, and anti-HIV drugs [6,7,8,9]. It has been reported that P-gp plays an important role in transporting lipid, endogenous opium peptide and naturally-occurring glucocorticoid cortisol, and regulating lipid metabolism [10,11].

**Figure 1:**
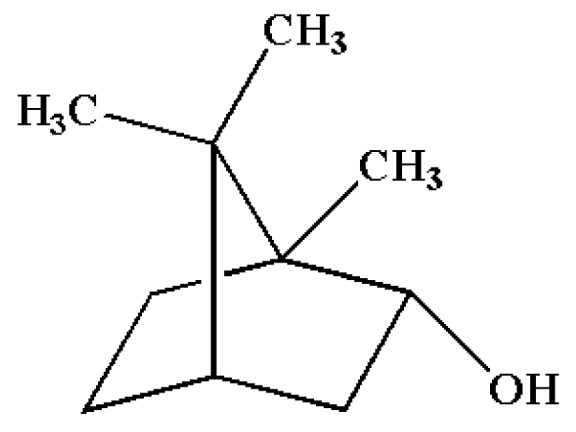
Chemical structure of borneol.

Borneol (Figure 1), Chinese materia medica monomer (molecular weight 154.24), is extracted from *Dryobalanops aromatica Gaertn f.* and *Blumea balsamifera DC*, and is widely used for the treatment of cardiovascular and cerebrovascular diseases in China. Previous studies showed that borneol was able to improve the permeability of BBB by a physiological process, enhance penetration and accumulation of some drugs into the brain such as tetramethylpyrazine phosphate, gastrodin, puerarin, geniposide, kaempferol and nimodipine, and increase the brain bioavailability of these drugs [12,13,14,15,16,17,18,19]. The mechanisms of borneol opened BBB focused on loosening the endothelial tight junctions, increasing the number and volume of pinocytosis, and decreasing the mdr1a and mdr1b expression in hippocampus and hypothalamus [20,21,22]. Although previous studies showed the clear effects of borneol on improving other drugs or compounds transportation to the brain parenchyma, the mechanisms are still not well known.

Because of the complexity of BBB control substances transport into the brain and the difficulty to explore the metabolisms *in vivo*, *in vitro* BBB models have been developed to study the screening substances according to their permeability across the BBB and related mechanisms. Compared with the *in vivo*, the principal advantages of the *in vitro* BBB models are the higher throughput capacity and the lower cost [23], *in vitro* BBB models also could unravel the complex cellular connections and molecular interactions that regulate the function and permeability of BBB [24]. In the present study, we established an *in vitro* BBB model comprised of rat BMECs and astrocytes to investigate the effects of borneol on the P-gp substrates transport through BBB as well as the intracellular mechanisms that regulate the effects of borneol on P-gp functions.

## 2. Results

### 2.1. Cell Characterization and Establishment of an in Vitro BBB Model

**Figure 2:**
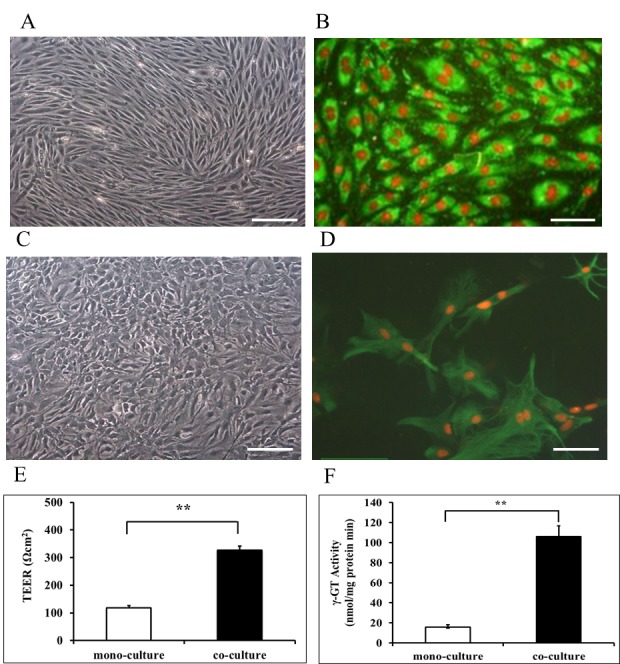
Cell characterization and establishment of the *in vitro* BBB model. (**A**) Representative image for primary rat BMECs, bar = 100 µm; (**B**) Representative vWF positive (green) and nucleus (red) immunofluorescence staining of BMECs, bar = 50 µm; (**C**) Representative image for primary rat astrocytes, bar = 100 µm; (**D**) Representative GFAP positive (green) and nucleus (red) immunofluorescence staining of astrocytes, bar = 50 µm; (**E**) TEER in mono-culture of BMECs and co-culture between BMECs and astrocytes; (**F**) γ-GT activity on BMECs. Data are expressed as mean + SD; ** *p* < 0.01 (*n* = 6 per group).

The primary rat BMECs presented a flat polygon-shaped phenotype and formed a monolayer characterized by being tightly packed and non-overlapping (Figure 2A). The BMECs were characterized by positive immunofluorescence staining with von Willebrand factor (vWF) antibody (Figure 2B). The primary astrocytes presented as star-shaped with numerous processes and formed layers of overlapping (Figure 2C), and were identified by positive glial fibrillary acidic protein (GFAP) immunofluorescence staining (Figure 2D). To test the functionality of the *in vitro* BBB model and the induction of tight junction complexes, the transendothelial electrical resistance (TEER) was measured. BMECs co-cultured with astrocytes showed a significant increase in TEER compared to mono-culture (*p* < 0.01) (Figure 2E). γ-Glutamyl transpeptidase (γ-GT) activity in isolated BMECs from co-culture and mono-culture was tested, γ-GT activity of BMECs in co-culture was seven-fold higher than that of mono-culture (Figure 2F).

### 2.2. Borneol Down-Regulated P-gp Efflux Function

**Figure 3:**
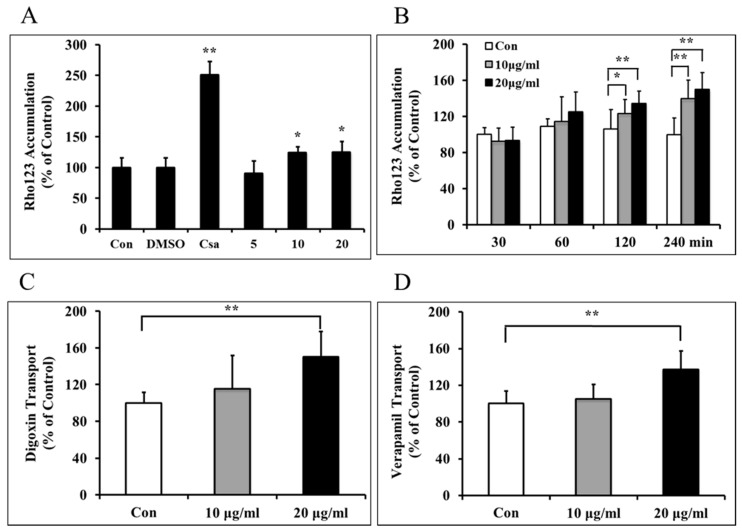
Effects of borneol on P-gp-mediated efflux function in BMECs. (**A**) Borneol increased the BMECs uptake of the P-gp substrate Rho123 in a dose-dependent manner. Con, control; DMSO, dimethyl sulphoxide; Csa, cyclosporin A; (**B**) Borneol increased cellular accumulation of Rho123 in a time-dependent manner; (**C**) Borneol enhanced P-gp substrate digoxin transport across the BBB *in vitro* model analyzed by high performance liquid chromatography (HPLC) at 4 h after treatment; (**D**) Borneol enhanced P-gp substrate verapamil transport across the BBB *in vitro* model analyzed by HPLC at 4 h after treatment. Data are expressed as mean + SD; * *p* < 0.05, ** *p* < 0.01 (*n* = 8 per group).

Cyclosporin A (CsA), as P-gp inhibitor, strongly enhanced Rhodamine 123 (Rho123) uptake by BMECs, and similarly borneol treatment enhanced the BMECs uptake of the P-gp substrate Rho123 in a dose-dependent manner (Figure 3A). Amounts of 10 μg/mL and 20 μg/mL borneol significantly increased cellular accumulation of Rho123 in a time-dependent manner by approximately 40% and 50% increase respectively at 240 min after treatment (Figure 3B). Amounts of 10 μg/mL and 20 μg/mL borneol were able to increase digoxin across BBB *in vitro* at 4 h after treatment, especially 20 μg/mL borneol enhanced by approximately 50% compared to control (*p* < 0.01) (Figure 3C). Similarly 10 μg/mL and 20 μg/mL borneol increased verapamil across BBB *in vitro*, especially 20 μg/mL borneol enhanced by approximately 37% compared to control (*p* < 0.01) (Figure 3D).

### 2.3. Effects of Borneol on mdr mRNA and P-gp Expression

**Figure 4:**
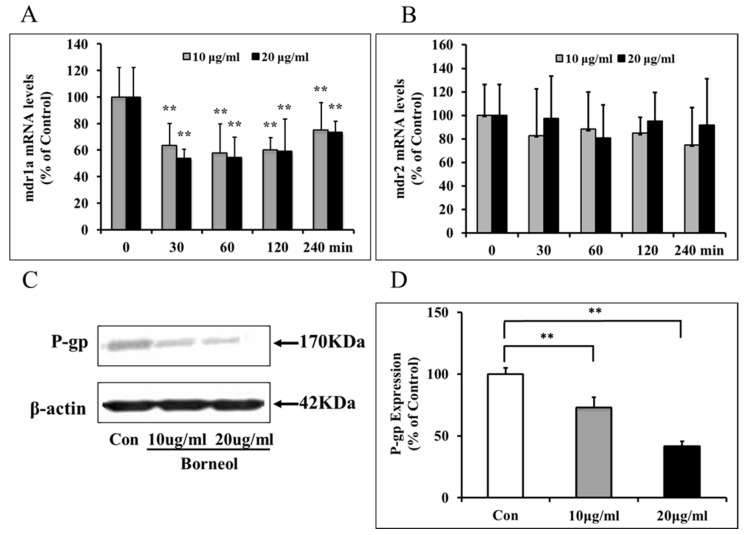
Effects of borneol on mdr mRNA and P-gp expression. (**A**) Borneol treatment decreased mdr1a mRNA expression in BMECs by a dose-dependent and time-dependent manner. Data are expressed as mean + SD; ** *p* < 0.01, *vs.* 0 min. (*n* = 8 per group); (**B**) Borneol did not change mdr2 mRNA levels of BMECs; (**C**) Representative P-gp expression by western blot on BMECs treated with borneol; (**D**) Quantitative data of P-gp expression. Data are expressed as mean + SD; ** *p* < 0.01 (*n* = 3 per group).

Borneol treatment decreased mdr1a mRNA expression in BMECs by a dose-dependent and time-dependent manner, and the mdr1a mRNA expression was minimum at 30 min to 1 h after treatment, then gradually went up at 2 to 4 h after treatment, but the levels were still lower than 0 min (Figure 4A). This indicated borneol could down-regulate mdr1a mRNA levels transiently and return to normal levels in a few hours. Borneol did not change mdr2 mRNA levels of BMECs (Figure 4B). Moreover, 10 μg/mL and 20 μg/mL borneol treatment decreased P-gp expression in BMECs, the reduction of P-gp expression were 27% and 58% compared to control group respectively at 4 h after treatment (Figure 4C,D).

### 2.4. Depressed P-gp Expression in BMECs by Borneol via a NF-κB Mediated Mechanism

**Figure 5:**
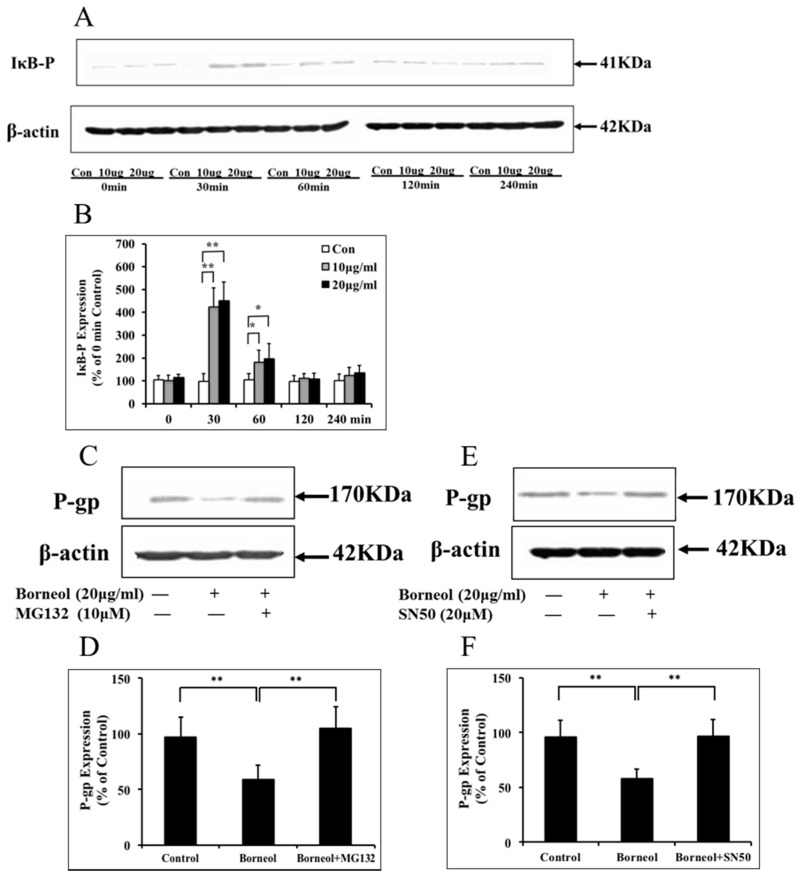
Measurement of NF-κB expression in BMECs. (**A**) Representative phosphorylated IκB expression by Western blot on BMECs treated with borneol up to 4 h; (**B**) Quantitative data of phosphorylated IκB expression; (**C**) Representative P-gp expression by Western blot on BMECs treated with borneol and borneol + MG132, respectively; (**D**) Quantitative data of P-gp expression; (**E**) Representative P-gp expression by Western blot on BMECs treated with borneol and borneol + SN50, respectively; (**F**) Quantitative data of P-gp expression. Data are expressed as mean + SD; * *p* < 0.05, ** *p* < 0.01 (*n* = 3 per group).

Phosphorylated expression of IκB was measured to detect whether the NF-κB signaling pathway involved in the regulation of P-gp expression in BMECs treated with borneol. 10 μg/mL and 20 μg/mL borneol significantly increased phosphorylation of IκB expression at 30 min after treatment transiently. The elevated phosphorylation of IκB returned to control levels within 120 min after treatment (Figure 5A,B). BMECs were pre-incubated with the specific NF-κB inhibitors, MG132 (10 μM) and SN50 (20 µM), for 1 h and treated with 20 μg/mL borneol for 4 h. Borneol treatment decreased P-gp expression in BMECs, while MG132 and SN50 obscured the borneol induced P-gp decreases (Figure 5C–F).

## 3. Discussion

Experimental results from this study showed borneol increased intracellular accumulation of Rho123, and enhanced P-gp substrates across the BBB *in vitro*, and also depressed mdr1a mRNA and P-gp expression. Furthermore, borneol could activate NF-κB and inhibition of NF-κB with MG132 and SN50 obscured the P-gp decreases induced by borneol. These data suggested that borneol depresses P-gp function in BMECs by a NF-κB signaling mediated mechanism in a BBB *in vitro* model.

The BBB represents a complex cellular system which controls transportation of substances between blood and brain and maintains brain microenvironment homeostasis. *In vivo* studies are limited because of the complexity of the BBB structure as well as the difficulty to explore the metabolisms and transport of substances. For these reasons, *in vitro* BBB models have been developed. Previous studies suggested that astrocytes are able to induce BBB properties of endothelial cells, endothelial cell mono-culture or endothelial cells and astrocytes co-culture models have become the most widespread *in vitro* BBB models. In endothelial cell mono-culture, endothelial cells are seeded on the filter membrane of Transwell, and the astrocyte conditioned medium induced BBB properties of endothelial cells, whereas in non-contact co-culture, astrocytes are cultured on the bottom of the wells [25]. In both these models, the endothelial cells lose their BBB characteristics over time. In general, *in vitro* models should gather and keep as many BBB characteristics as possible. In this study, we established an *in vitro* BBB model comprising of rat BMECs and astrocytes, in which model astrocytes are cultured at the opposite sites of the polyethylene terephthalate (PET) membrane of Transwell with close contact with BMECs. The morphology of the primary BMECs in the present study was a typical feature of endothelial cells of BBB (Figure 2A), and the positive immunofluorescence staining with vWF antibody proved their endothelial origin and purity (Figure 2B). Previous studies demonstrated that cultured BMECs *in vitro* dedifferentiate very quickly and lose their specific characteristics of BBB [26,27]. Tight junctions between BMECs play an essential role to maintain the BBB function and properties [28]. The TEER correlated with the establishment of a tight junction has already been determined in MDCK cultures study [29]. The TEER in our established BBB *in vitro* model reached high levels, there was a very significant difference between BMECs co-culture with astrocytes and BMECs mono-culture (Figure 2E). Other studies also suggested astrocytes could increase γ-GT activity of vascular endothelial cells [30,31]. Our results agreed with these studies and showed significant increase of γ-GT activity in co-culture with closed contacts of the BMECs and astrocytes (Figure 2F). Compared with the endothelial cell mono-culture or endothelial cells and astrocytes no-contact co-culture models, the *in vitro* BBB model established in our study had special BBB characteristics of the higher TEER that indicated the paracellular permeability and BBB integrity, and higher expression of γ-GT, one of the key enzymes of the endothelial cells of BBB. In the present study, we successfully established the *in vitro* BBB model comprised of BMECs and astrocytes with the special BBB characterization.

Rho123 is a fluorescent dye which can be removed from the cells by P-gp, and P-gp function can be evaluated by Rho123 efflux assay *in vitro* and *in vivo* [32,33]. Therefore, an increased cellular accumulation of Rho123 is generally considered as the marker of diminished P-gp efflux function. However, it was interesting that borneol was able to decrease accumulation of Rho123 in BMECs by transporter mechanisms (Figure 3A,B). Also borneol could improve the known P-gp substrates, verapamil and digoxin, transport through the *in vitro* BBB model (Figure 3C,D). These data suggested that borneol could down-regulate P-gp efflux function and enhance P-gp substrates transport across BBB.

Previous studies demonstrated that brain concentrations of pharmacological agents were dramatically increased in mdr1a-deficient mice when compared to wild-type mice [34]. P-gp expressed on the luminal membrane of BMECs, which encoded by mdr1a, confers multidrug resistance to different chemotherapeutic agents [35]. Several extracellular stimulants have been reported to enhance MDR1 mRNA expression such as serum, mitogen stimulation, heavy metals, heat shock, and so on [36]. Our study demonstrates that borneol could down-regulate mdr1a mRNA levels at 30 min to 4 h after treatment (Figure 4A), and borneol cannot change the levels of mdr2 mRNA. Rodent mdr1a is predominant gene expressed in BMECs that selectively confers multidrug resistance. We confirmed the P-gp expression was suppressed by borneol at 4 h after treatment, probably because mdr1a mRNA expression was inhibited by borneol (Figure 4C,D). These data suggest that borneol could down-regulate P-gp efflux function by suppressing mdr1a mRNA and P-gp expression.

Recent studies have shown that factors released from brain parenchyma after ischemic stroke could potentially influence P-gp expression. H_2_O_2_ can increases P-gp expression in primary rat BMECs mediated by increased transcription and various signaling pathways, such as extracellular signal-regulated kinases (ERK) 1/2, protein kinase c (PKC), stress-activated protein kinases (SAPK) and protein kinase B (AKT) [37]. NF-κB is one of important pathways to modulate P-gp expression [38], previous studies also indicate that PI3-kinase can regulate human MDR1 expression via NF-κB [36], of which the binding site was located in the MDR1 promoter region upstream from the MDR1 transcription starting site [36,39]. It was reported that NF-κB could regulate P-gp expression dependently in the liver and kidney [40,41]. The possible involvement of the NF-κB signaling pathway in P-gp expression in rat BMECs treated by borneol was tested. In the present study, we demonstrated that borneol depressed mdr1a mRNA and P-gp expression, and borneol activated NF-κB signaling transiently which peaked at 30 min and returned to control levels within 120 min after treatment (Figure 5A,B). Moreover, blockade of NF-κB by IκB degradation with MG132 or NF-κB translocation with SN50 could obscure the borneol induced P-gp decreases (Figure 5C–F). It is possible that MG132 works more directly by blocking P-gp degradation and SN50 inhibits NF-κB translocation to the nucleus. In addition, there are reports that NF-κB has an inhibitory effect on P-gp expression in rat BMECs [38], and it can provide negative regulation of P-gp [42,43].

There are several caveats and limitations in this work. First, although the *in vitro* model was very similar to BBB *in vivo* and more convenient to study transport protein functions of BBB and related mechanisms, it is still a little different from the *in vivo* condition. Second, we only transiently treated the BBB *in vitro* with borneol for 4 h, actually this is a short treatment time window. We did not monitor the long term effects of borneol on BBB function because of the culture limitation of *in vitro* BBB model. Further experiments to measure the multifactorial pathways of borneol on other substances especially Chinese materia medica across BBB *in vivo* would be clinically important.

## 4. Experimental Section

### 4.1. Materials

Dulbecco’s minimum essential medium (DMEM), Type II collagenase, DNAse I were obtained from Invitrogen (Carlsbad, CA, USA). Collagenase/dispase and endothelial cell growth factor were purchased from Roche Molecular Biochemicals (Indianapolis, IN, USA). Fetal bovine serum (FBS) was obtained from Hyclon (Lgan, UT, USA). Bicinchoninic acid (BCA) protein assay reagent kit was obtained from Pierce (Rockford, IL, USA). Transwell inserts were purchased from Costar (Bethesda, MD, USA). Anti-P-gp monoclonal antibody was purchased from Calbiochem (La Jolla, CA, USA). The antibody to phospho-IκB, the horseradish peroxidase-conjugated secondary antibodies, and enhanced chemiluminescent (ECL) Western blotting detection reagents were purchased from Santa Cruz Biotechnology (Santa Cruz, CA, USA). The antibody to β-actin was from Sigma (St. Louis, MO, USA).

### 4.2. Isolation and Culture of Rat Brain Microvascular Endothelial Cells

A modified method described in the literature was used to isolate rat BMECs [44]. Briefly, 1-month-old Wistar rat cortices were dissected free of meninges and cut into small pieces on ice, then digested in a mixture of type II collagenase (0.1%) and DNAse (10 U/mL) in DMEM for 1 h at 37 °C. The pellet was separated by centrifugation in 15% dextran/PBS (4500 rpm, 20 min) and further digested in 1 mg/mL collagenase/dispase and DNAse (10 U/mL) in DMEM for 1 h at 37 °C. After digestion, the pellet was re-suspended and layered over 50% continuous Percoll gradient. After centrifugation, microvascular fragments and cells were collected and seeded on gelatin coated tissue culture plates or culture flasks. BMECs-specific medium consisted of DMEM supplemented with 20% FBS, 150 μg/mL endothelial cell growth factor, 100 μg/mL heparin, 2 mM l-glutamine, 100 U/mL penicillin and 100 μg/mL streptomycin. BMECs were confirmed by immunofluorescence staining for vWF and fluorescein isothiocyanate (FITC)-conjugated secondary antibody.

### 4.3. Isolation and Culture of Rat Astrocytes

Astrocytes were isolated from the cerebral cortices of 1 to 2-day-old Wistar rat pups according to the previous methods [45]. The cerebral cortices were dissected free of meninges and cut into small pieces, then digested in 0.25% trypsin/0.02% EDTA in PBS for 5 min at 37 °C. The homogenized tissue was forced through a 100 µm nylon sieve. After centrifugation, the pellets were re-suspended in DMEM (supplemented with 10% FBS, 2 mM l-glutamine, 100 U/mL penicillin and 100 μg/mL streptomycin) and plated into 75 cm^2^ tissue culture flasks. At 8 days of culture, the confluent astrocytes were shaken at 260 rpm for 18 h at 37 °C in order to eliminate the contaminating microglia and oligodendroglia. Astrocytes were confirmed by immunofluorescence staining for GFAP and FITC-conjugated secondary antibody.

### 4.4. The Establishment of an in Vitro BBB Model

The *in vitro* BBB model consisted of rat BMECs and astrocytes grown on two-sides of PET membrane of Transwell (Figure S1). Briefly, astrocytes (1 × 10^5^/cm^2^ cells) were cultured on the opposite side of collagen-coated PET membrane of Transwell (Costar, pore size 0.4 mm; diameter 12 mm; insert growth area 1 cm^2^). When astrocytes grew about 60% confluent monolayer, BMECs (2 × 10^5^/cm^2^ cells) were seeded on the upper side of the PET membrane in the apical chamber.

### 4.5. Measurement of Transendothelial Electrical Resistance (TEER)

The *in vitro* BBB model integrity was assessed by TEER measurement, according to previous methods [46]. The electrical resistance across the endothelial cells was measured by an ERS (Millicell, USA) (Figure S1). The electrical resistance of blank membranes of Transwell was subtracted from membranes with cells. TEER was shown as Ωcm^2^.

### 4.6. Measurement of γ-GT Activity in BMECs of the in Vitro BBB Model

γ-GT activity of BMECs was measured as in previously described methods [47]. In brief, the BMECs were collected from the PET membrane of Transwell and sonicated for 15 s at 20 W with a Vibracell TM 75022 ultrasonic processor. γ-l-Glutamyl-*p*-nitroanilide and glycylglycine were used as substrates and the production of p-nitroaniline is considered as γ-GT activity. The absorbance of p-nitroaniline was measured at 540 nm and protein concentration was determined using the BCA assay.

### 4.7. The Effects of Borneol on Rho123 Accumulation in BMECs

Rho123 efflux assay was used to measure the activity of P-gp in BMECs according to previous methods [32]. BMECs grown to confluency in 24-well plates were treated with 5 μg/mL, 10 μg/mL and 20 μg/mL borneol, DMSO, CsA for 2 h, or with 10 μg/mL and 20 μg/mL borneol for different times (30 min, 1 h, 2 h, and 4 h). Then BMECs were exposed to 5 μmol/L Rho123 in DMEM for 90 min. After incubation with Rho123, BMECs were washed with ice-cold PBS and solubilized in 1% NaOH. Fluorescence of Rho123 was measured with emission wavelength at 535 nm and excitation wavelength at 485 nm using a fluorescence spectrophotometer (BioTek, Winooski, VT, USA). Rho123 levels were normalized to total cell protein and shown as nmol Rho123/mg protein.

### 4.8. The Effects of Borneol on P-gp Substrates Transport through the in Vitro BBB Model

To validate the effects of borneol on P-gp substrates transport through the *in vitro* BBB model, verapamil and digoxin, as known P-gp substrates, were measured. The medium from the apical compartments was replaced with 0.5 mL BMECs-specific medium including tested compounds (digoxin, digoxin + 10 μg/mL borneol, digoxin + 20 μg/mL borneol, verapamil, verapamil + 10 μg/mL borneol, verapamil + 20 μg/mL borneol) and the basal compartment with 1.5 mL BMECs-specific medium. At 4 h after treatment, medium was collected from the basal compartment for HPLC analysis. The ratio of the permeability concentration (apical to basal compartment) was then calculated.

### 4.9. Real-Time RT-PCR Analysis

Mdr gene expression in BMECs treated with borneol was determined by Real-time RT-PCR analysis as described earlier [48]. Briefly, total RNA was purified from BMECs treated by borneol with Trizol Reagent (Invitrogen, Carlsbad, CA, USA) according to the manufacture’s instruction. cDNAs of the mdr1a, mdr2 and glyceraldehyde-3-phosphate dehydrogenase (GAPDH) were quantified using SYBR Green PCR Master Mix reagent kits (Applied Biosystems, Foster City, CA, USA) performed with the ABI PRISM 7300 Sequence Detection System. The following sequences of mdr1a, mdr2, and GAPDH were designed with Primer Express 2.0 (Applied Biosystems, Branchburg, NJ, USA) and used in present study: mdr1a forward, 5’-GCAGGTTGGCTGGACAGATT-3’; mdr1a reverse, 5’-GGAGCGCAATTCCATGGATA-3’; mdr1a probe, 5’-FAM-CCG CCA GAG TTC CCA GCA GCA TG-TAMRA-3’; mdr2 forward, 5’-AGTTCACGGGCGCATCAA-3’; mdr2 reverse, 5’-AAAAGACACTGGTGGCACGTT-3’; mdr2 probe, 5’-FAM-CAT CAA GTT CAT TGG TTT CCA CAT CCA GC-TAMRA-3’; GAPDH (an internal reference gene) forward, 5’-CCCCCAATGTATCCGTTGTG-3’; GAPDH reverse, 5’-TAGCCCAGGATGCCCTTTAGT-3’; GAPDH probe, 5’-FAM-TGC CGC CTG GAG AAA CCT GCC-TAMRA-3’. Comparative threshold cycle (*C*_t_) method was used to analyze data and *C*_t_ values from mdr1a and mdr2 were normalized with the values from corresponding GAPDH reactions.

### 4.10. Western Blot Analysis

Western blot analysis was performed following the standard method [49]. Briefly, homogenates of BMECs treated with borneol were prepared in lysis buffer and protein concentrations were determined using the BCA protein assay, and equal amounts of protein were mixed with 2× SDS-PAGE sample loading buffer. After heating at 95 °C for 5 min, proteins were loaded to 7.5% (for P-gp) or 12% (for IκB) tris-glycine gel and transferred to polyvinylidene difluoride membrane (Bio-Rad, Hercules, CA, USA) electrophoretically. Incubation was carried out with anti-P-gp (1:1000) antibody or anti-phosphospecific antibody to IκB (1:200) at 4 °C overnight and then with horseradish peroxidase-labeled secondary antibodies (1:2000) for 1 h at room temperature. Antibody positive bands representing the proteins of interest were visualized using ECL Western blot detection reagents. The band densities were measured by Quantity One software using Bio Imaging Systems (GeneGenius, SynGene, Cambridge, UK).

### 4.11. Statistical Analysis

Data were expressed as mean + SD. All measurements were assessed with ANOVA, followed by Tukey–Kramer tests or Independent-Samples *t* test. Differences with *p* < 0.05 or *p* < 0.01 were considered statistically significant.

## 5. Conclusions

Taken together, we have provided evidence that borneol can down-regulate P-gp efflux function, and decrease P-gp mRNA and protein expression in BMECs transiently by a NF-κB signaling mediated mechanism in a BBB *in vitro* model. This finding identifies the possibility that borneol could provide a narrow time window for those usually impermeable drug P-gp substrates (many chemotherapeutics and Chinese materia medica) to selectively transport across BBB and enter the brain. In addition, this may contribute in identifying pharmacological targets that transiently reduce P-gp-mediated drug efflux function with minimal side effects.

## Supplementary Materials

Supplementary materials can be found at http://www.mdpi.com/1422-0067/16/11/26051/s1.
