# Supplementary material for: Borneol Depresses P-Glycoprotein Function by a NF-κB Signaling Mediated Mechanism in a Blood Brain Barrier in Vitro Model
Source: Int J Mol Sci. 2015 Nov 18;16(11):27576–88. doi: 10.3390/ijms161126051 (PMC4661909; doi:10.3390/ijms161126051)
Supplement: Supplementary file 1 [file ijms-16-26051-s001.pdf]

Supplementary Materials: Borneol Depresses P-Glycoprotein Function by a NF-κB Signaling Mediated Mechanism in a Blood Brain Barrier *in Vitro* Model

Xiang Fan, Lijuan Chai, Han Zhang, Yuefei Wang, Boli Zhang and Xiumei Gao

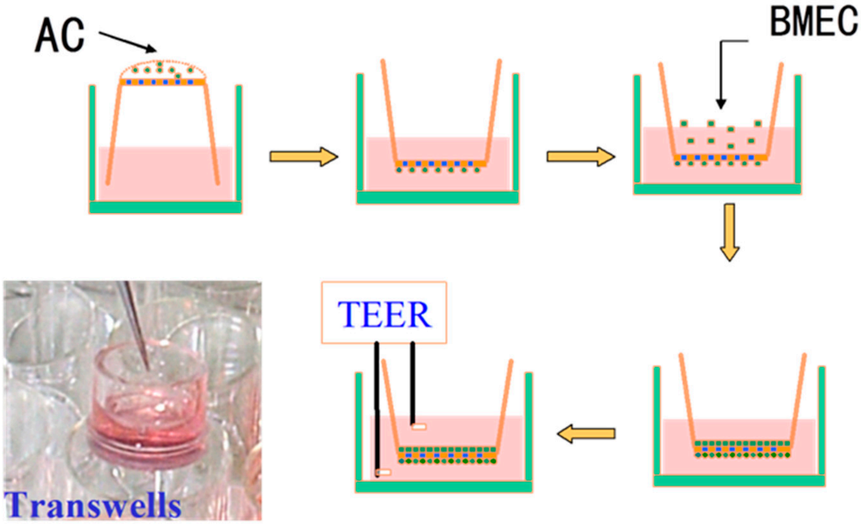

Figure S1. The flow chart of *in vitro* blood brain barrier model establishment. BMEC, brain microvascular endothelial cells; AC, astrocytes; TEER, transendothelial electrical resistance.
